# Impact of the corneal epithelium on the corneal power using 3D raytracing with OCT data

**DOI:** 10.1016/j.zemedi.2025.01.002

**Published:** 2025-02-11

**Authors:** Achim Langenbucher, Nóra Szentmáry, Alan Cayless, Peter Hoffmann, Jascha Wendelstein

**Affiliations:** a Department of Experimental Ophthalmology, Saarland University, Homburg/Saar, Germany; b Dr. Rolf M. Schwiete Center for Limbal Stem Cell and Aniridia Research, Saarland University, Homburg/Saar, Germany; c Department of Ophthalmology, Semmelweis-University, Budapest, Hungary; d School of Physical Sciences, The Open University, Milton Keynes, United Kingdom; e Augen- und Laserklinik Castrop-Rauxel, Castrop-Rauxel, Germany; f Department of Ophthalmology, Johannes Kepler University Linz, Austria; g Department of Ophthalmology, LMU Klinikum, Munich, Germany

**Keywords:** Corneal power, Corneal epithelium, 3-surface corneal model, Raytracing, Best focus

## Abstract

**Purpose:**

To study the effects of corneal imaging and focusing using a raytracing simulation with 2 and 3 surface corneal models based on customized surface representations of corneal tomography data .

**Methods:**

Raytracing simulation using surface data for the epithelium (S1), stroma (S2) and endothelium (S3) extracted from MS-39 anterior segment tomographer CSV export files. Customized surface representations were derived using Gaussian Process Predictors, and rays traced through the cornea and a 3.5 mm aperture stop located 3.66 mm behind the corneal apex. 4 clinical examples were evaluated: A) after hyperopic LASIK, B) after myopic LASIK, C) keratoconus, and D) after PRK with postoperatively developed Salzmann nodules.

**Results:**

The raytracing based bundle focus and wavefront focus distances of the 2 surface (S1 and S3) and 3 surface cornea models (S1, S2 and S3) were comparable, whereas the paraxial focus derived from a 1 surface cornea (S1), 2 (S1 and S3) or 3 surface cornea (S1, S2 and S3) using floating best fit sphere representations for S1, S1 and S3 showed systematically lower / higher focal distance with B) / C) indicating an overestimation / underestimation of corneal power with paraxial calculations.

**Conclusions:**

The clinical examples in this study exhibited only minor differences between the mono- and dual layer cornea models. We recommend verification in a larger clinical study. Three surface corneal raytracing models could be of clinical relevance in intraocular lens calculations and LASIK ablation nomograms, offering potential improvements over paraxial calculations especially in cases with surface irregularities.

## Background

There has previously been some controversial discussion regarding the effect of the corneal epithelium on the refractive power of the cornea [1], [2]. Especially in the case of pathologies such as corneal ectasia with keratoconus, keratoglobus, or pellucide marginal degeneration, or in eyes with a history of corneal surgery such as PRK, LASIK, or pterygium excision, there are arguments that an inhomogeneous epithelial layer thickness or local variations in the refractive index [3] might have an appreciable refractive effect and that this should be considered e.g. in intraocular lens power calculation, or laser vision correction ablation nomograms.

The literature contains several studies regarding the refractive index of the tear film layer, the corneal epithelium, and the stroma [4], [5], [6]. In general, these studies find that the refractive index of the tear film (n_T_ = 1.336) is similar to that of water (1.333). The epithelium shows the highest refractive index (n_E_ = 1.40), and the corneal stroma is characterized by a refractive index similar to that stated in various schematic eye models (n_S_ = 1.376) [4], [5].

The latest generation of high-resolution anterior segment analyzers are capable of measuring the interface between the epithelium and the stroma in addition to the corneal front and back surfaces and can export height data for all 3 corneal surfaces as data maps with a resolution sufficient to be used directly for raytracing. Such devices include the MS-39 (CSO, Scandicci, Italy), the Casia 2 (Tomey, Nagoya, Japan) and the Anterion (Heidelberg engineering, Heidelberg, Germany). The raytracing results [7] provide some understanding of the impact of the corneal epithelium on corneal power and corneal imaging properties by comparing the data with the results of paraxial calculations based on a 2 surface (monolayer cornea with front surface (epithelium) and back surface (endothelium)) or a 3 surface model (dual layer cornea with epithelium and stroma).

Raytracing generally requires a closed description and representation of surfaces [1], [6], [8], [9], [10], [11], [12]. Tomographers export surface data as point clouds describing the surface at discrete surface points only [13]. There are several options for converting point cloud data to a closed surface representation, including interpolation, where the surface follows all datapoints in the point cloud, and surface approximations which are smoother as the surface is not constrained to follow all datapoints in the point cloud [13]. Since measurement data from OCT are quite noisy, interpolating schemes (e.g. subdivision schemes [9]) which follow each datapoint in the dataset risk generating large variations in the local slope of the surface, making the raytracing results unreliable. Therefore, surface approximations might show superior performance when applied to OCT corneal measurement data.

The **purposes of this study** were

• to describe a concept for raytracing through a two-layer cornea based on refractive surface models derived from point cloud data from a modern anterior segment OCT tomographer,
• to calculate the best focus and characteristic performance metrics and to compare the focus position with the result of a simple paraxial calculation, and
• to show the applicability of raytracing using selected clinical examples having corneal pathologies or with a history of corneal surgery where some variation in the epithelium layer thickness would be expected.

## Methods

**Figure 1:**
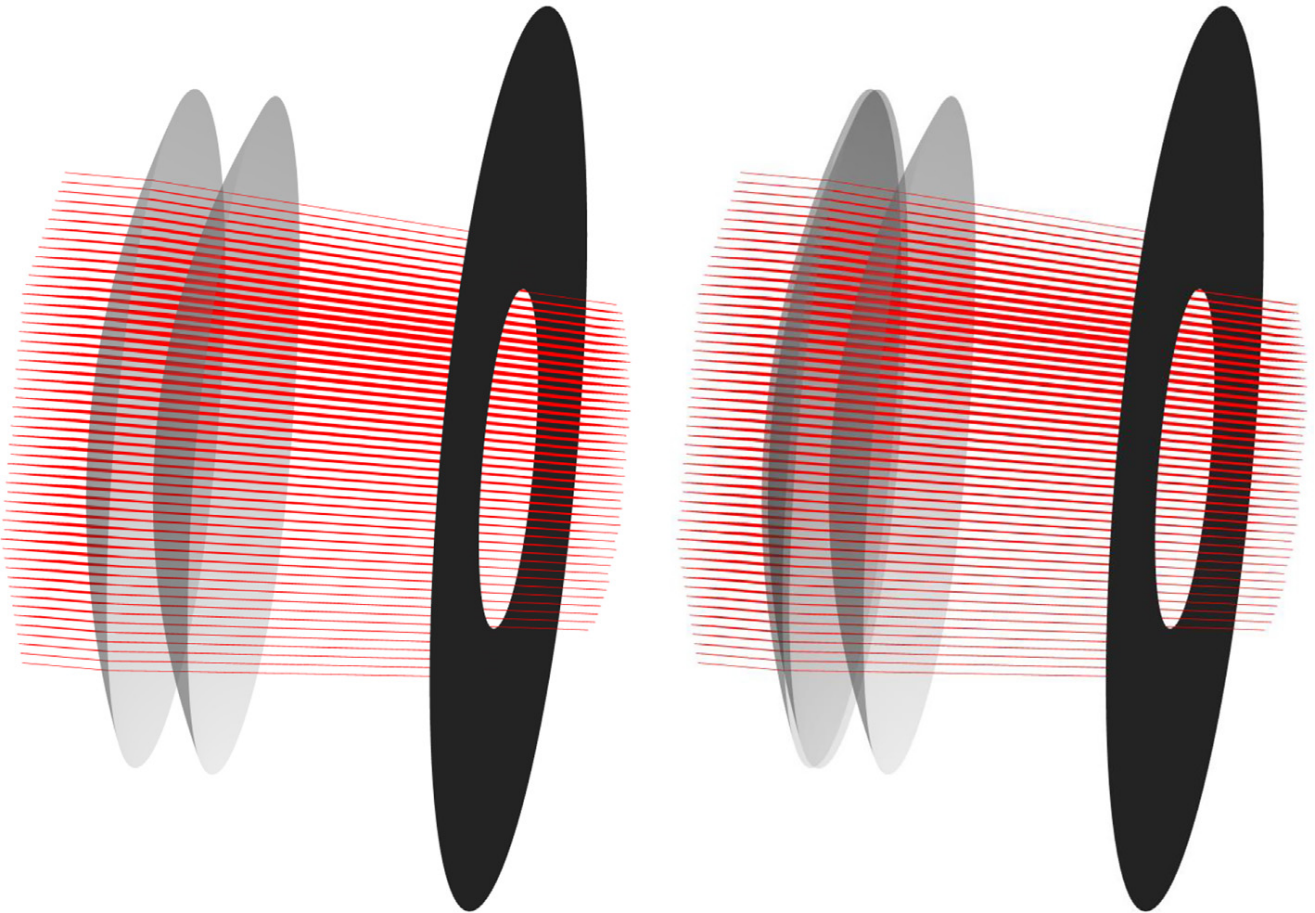
Optical bench setups used for raytracing: The left diagram shows the setup is for the cornea represented in terms of 2 refractive surfaces (corneal front and back surface), and the diagram on the right shows the cornea represented in terms 3 surfaces (corneal epithelium, corneal stroma, and corneal endothelium). The aperture stop (3.5 mm in diameter) was placed at 3.66 mm behind the corneal front apex. A subset of rays (N = 500) are plotted in red to aid visualization of the setup.

In this simulation study a raytracing setup including the cornea and an aperture was implemented. Corneal measurement data from a high resolution anterior segment analyzer (MS-39, CSO, Scandicci, Italy) were exported as CSV data files. Each exported file contains curvature and power data grid data for the surface height (epithelium, stroma and endothelium) organized in a cylindrical coordinate system for 256 equidistant semimeridians and 30 radial distances (from 0.2 to 6 mm distance from the center in equidistant steps of 0.2 mm). These grid data for the 3 refracting surfaces were extracted and cropped to a central region of 6 mm in diameter, producing data matrices with dimensions 15 x 256 for surface S1 (corneal epithelium), surface S2 (corneal stroma), and surface S3 (corneal endothelium). A 3.5 mm diameter aperture located coaxially 3.66 mm behind the corneal front surface (according to the schematic model eye of Liou & Brennan [14]) was used to represent the pupil. A ray bundle consisting of 10,000 collimated rays organized in an equidistant hexagonal grid of diameter 4.5 mm were initialised from a plane located 1 cm in front of the cornea. The refractive index of air was taken as that of vacuum, (n_V_ = 1.0) and literature data were used for the refractive indices of the epithelium (n_E_ = 1.40), the stroma (n_S_ = 1.376), and the aqueous humour (n_A_ = 1.336) [1], [4], [5]. Two scenarios were considered for raytracing: a 2 surface model of the cornea using data of S1 and S3 with refractive indices n_V_ in front of S1, n_S_, between S1 and S3, and n_A_ behind S3; and a 3 surface model of the cornea using data of S1, S2 and S3 with refractive indices n_V_ in front of S1, n_E_ between S1 and S2, n_S_, between S2 and S3, and n_A_ behind S3. Fig. 1 depicts the optical setups for the 2 surface model (left) and the 3 surface model (right) of the cornea together with the aperture stop and some indicator rays for better visualization (number of rays: 500).

Raytracing was implemented in MATLAB (Matlab version 2022b, The Math Works, Natick, USA). Prior to the raytracing, we fitted floating best fit spheres (BFS) to S1, S2, and S3 within the central 6 mm zone in terms of minimizing the root-mean-squared fit error, recording the mean radii of curvature R1, R2, and R3 and the centers of the spheres. The apices were extracted from these centers and the radii of curvature, and the radii and apices used for the paraxial calculations. These paraxial calculations (used as a reference for the raytracing results) included the refractive power (P) and focal distance (F, always with respect to the corneal front apex) for keratometry (PK and FK, for a 1 surface cornea using a keratometer index of n_K_ = 1.332) and the refractive powers for a 2 surface (P2 and F2) and the 3 surface cornea (P3 and F3).

We then used a Gaussian Process Predictor to define a closed representation for the 3 surfaces S1, S2, and S3 [15], [16], [17]. This Gaussian Process Predictor is known to yield a smooth, differentiable model of the corneal surfaces suitable for raytracing [17], [18]. The collimated ray bundle is clipped at the aperture stop after tracing through the 2 (2 surface model) or 3 (3 surface model) corneal surfaces. This clipped ray bundle behind the aperture stop is considered in order to find the best focus in terms of smallest root-mean-squared ray scatter (ray bundle focus FRB2 and FRB3 for the 2 and 3 surface model), and the focus in terms of the smallest root-mean-squared wavefront error (wavefront focus FWF2 and FWF3 for the 2 and 3 surface model). For this focus search, we implemented a nonlinear iterative SQP algorithm [10], [19], [20], [21], [22] initialized with the position of the paraxial keratometry focus FK. The stopping criteria were 1500 iterations, a step tolerance of 1e-12, and a function tolerance of 1e-12 [10], [21], [22].

For presenting the clinical examples, in addition to the corneal focus positions (FK, F2, F3, FRB2, FRB3, FWF2, and FWF3, all in mm), we also calculated the root mean squared ray scatter (in microns) and the root mean squared wavefront error (in microns) for the focus positions derived from raytracing (FRB2, FRB3, FWF2, and FWF3). To better understand the impact of the epithelial layer on the refraction and imaging properties of the cornea, 4 characteristic clinical examples are considered in this paper:

A) A tomography measurement of the right eye of a 45 years old male patient 8 years after laser vision correction (LASIK) for hyperopia (correction: 3.5 dioptres sphere and 0.75 dioptres cylinder) and a standard refraction-based ablation profile with an optical zone of 6.5 mm,
B) A tomography measurement of the left eye of a 41 years old female patient 6 years after laser vision correction (LASIK) for myopia (correction: -6.0 dioptres sphere and 0.5 dioptres cylinder) and a standard refraction-based ablation profile with an optical zone of 6.8 mm,
C) A tomography measurement of the right eye of a 36 years old male patient with a keratoconus 3 years after corneal crosslinking (with riboflavin, Dresden protocol), and
D) A tomography measurement of the left eye of a 40 years old female patient 4 years after Photorefractive Keratectomy (PRK) for surface smoothening (optical zone: 7.0 mm) showing epithelial irregularities and a development of Salzmann nodules.

**Figure 2:**
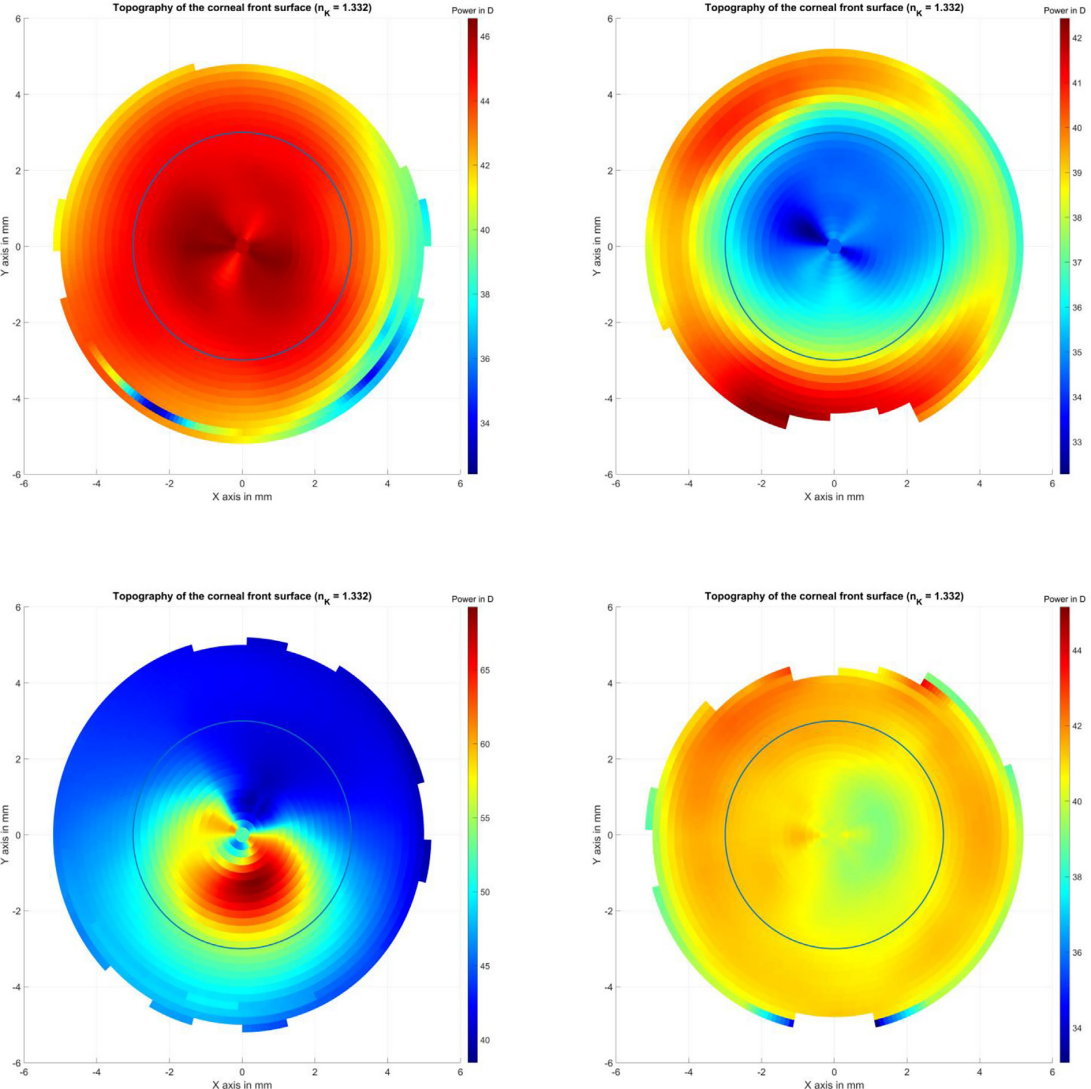
Corneal topographies of the 4 clinical examples shown in the paper: upper left graph: situation after hyperopic refractive laser surgery (case A), upper right graph: situation after myopic refractive laser surgery (case B), lower left graph: situation with keratoconus (case C), and lower right graph: situation after laser ablation of the surface with photorefractive keratectomy and subsequent Salzmann nodule development (case D). Corneal power was derived from axial corneal radius data using a keratometer index of n_K_ = 1.332. The circles in all graphs indicate the region of interest (diameter of 6 mm) used for the surface representation of the 3 corneal surfaces when using a Gaussian Process Predictor.

The corresponding corneal power profiles converted from axial front surface radius to dioptres using n_K_ are shown in Fig. 2 for the clinical cases A), B), C), and D).

## Results

**Figure 3:**
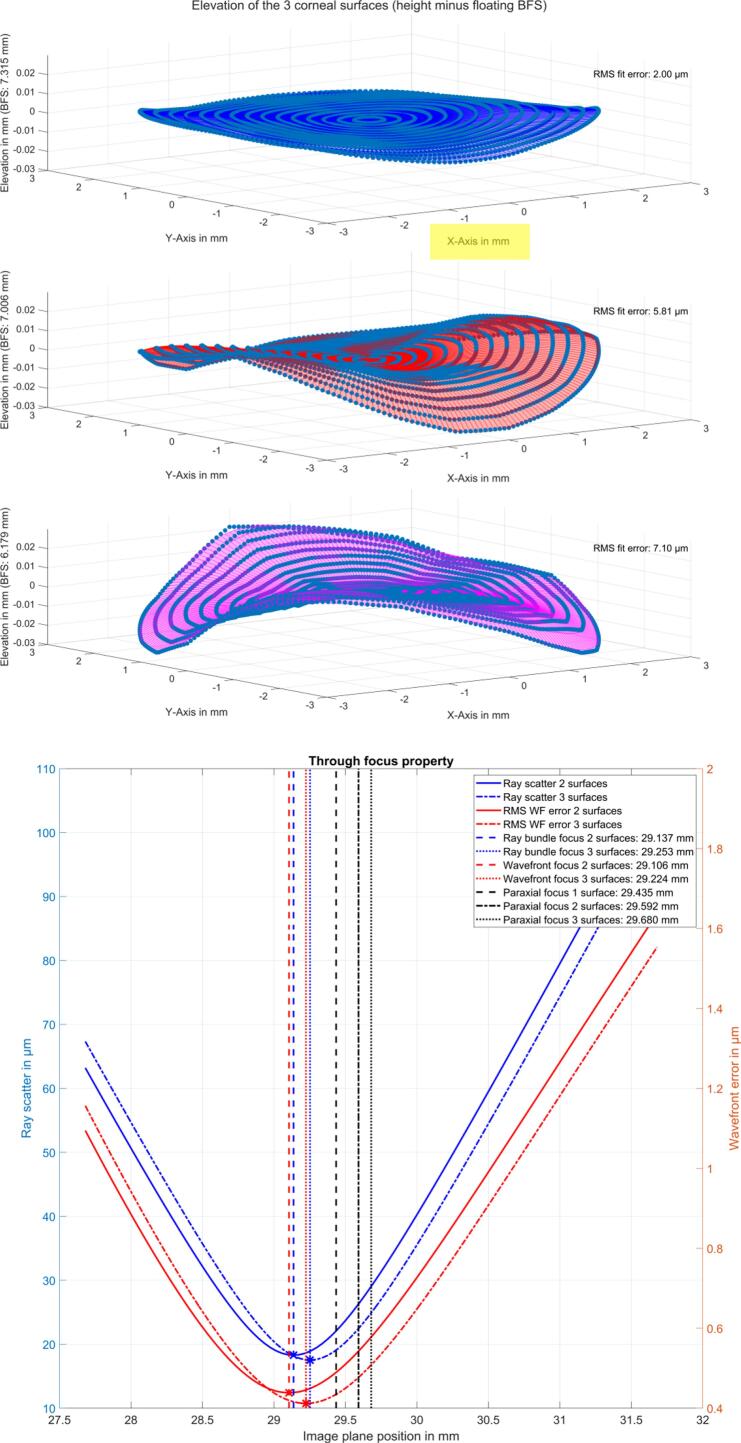
Clinical case A with a situation after hyperopic refractive laser surgery (LASIK). Upper three plots: Surface elevation as the height difference between the measurement data (filled dots) and the best fit sphere BFS is plotted together with the Gaussian Process (GP) surface representation used for raytracing (semitransparent surface) for the corneal epithelium (surface S1, upper plot), corneal stroma (surface S2, middle plot), and corneal endothelium (surface S3, lower plot). The root mean squared fit error of the GP surface representation is shown in the legend of each plot. Lower graph: Through focus performance (centered to the paraxial focus of keratometry with a range of ±2 mm) of the root mean squared ray bundle scatter (blue lines, scale on the left) and the root mean squared wavefront error (red lines, scale on the right) is shown for the corneal model with 2 surfaces (surface S1 and S3, solid line) and with 3 surfaces (surfaces S1, S2, and S3, dashdotted line) together with the respective ray bundle focus and the wavefront focus. The black dashed, dashdotted and dotted lines indicate the paraxial focus with a 1 surface cornea model (keratometric with S1, keratometer index n_K_ = 1.332), a 2 surface cornea model (S1 and S3), and a 3 surface cornea model (S1, S2, and S3) respectively.

**Figure 4:**
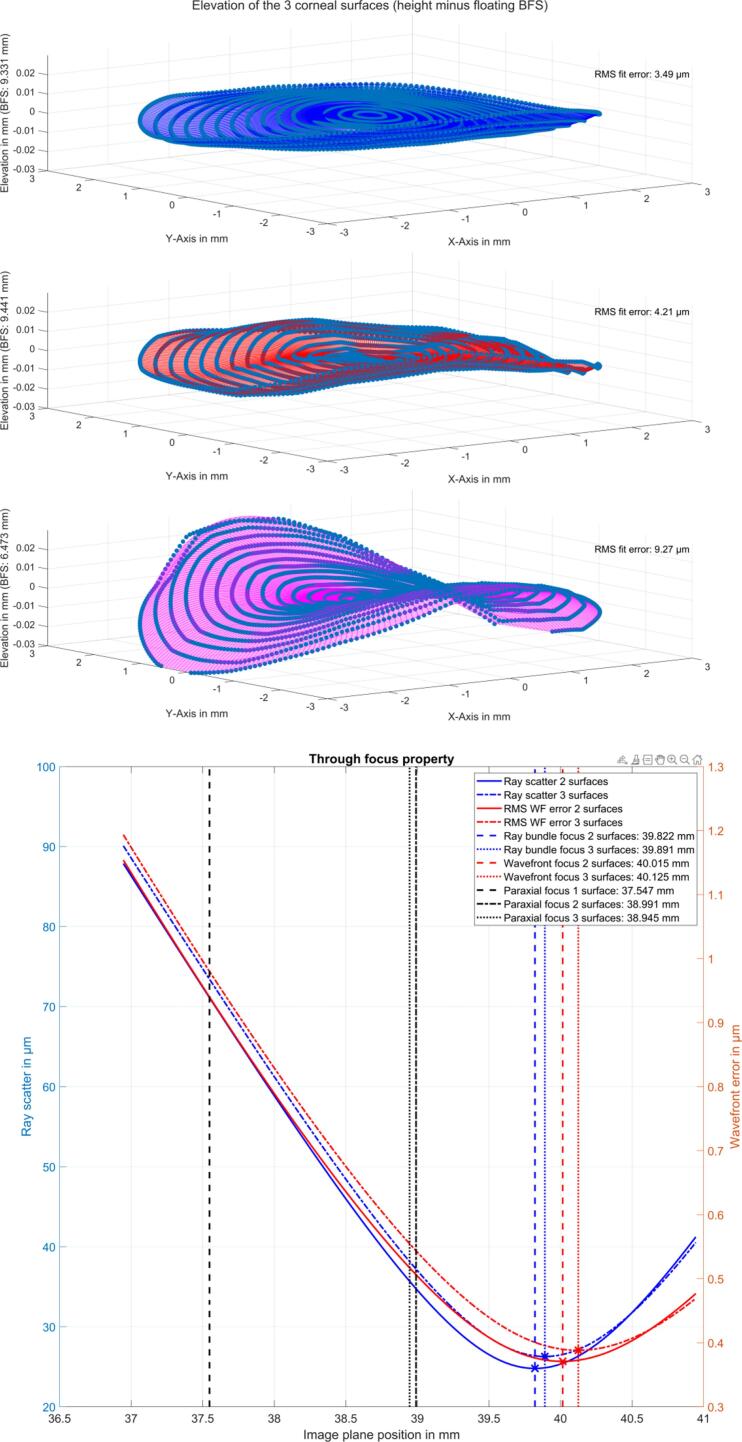
Clinical case B with a situation after myopic refractive laser surgery (LASIK). Upper three plots: Surface elevation as the height difference between the measurement data (filled dots) and the best fit sphere BFS is plotted together with the Gaussian Process (GP) surface representation used for raytracing (semitransparent surface) for the corneal epithelium (surface S1, upper plot), corneal stroma (surface S2, middle plot), and corneal endothelium (surface S3, lower plot). The root mean squared fit error of the GP surface representation is shown in the legend of each plot. Lower graph: Through focus performance (centered to the paraxial focus of keratometry with a range of ±2 mm) of the root mean squared ray bundle scatter (blue lines, scale on the left) and the root mean squared wavefront error (red lines, scale on the right) is shown for the corneal model with 2 surfaces (surface S1 and S3, solid line) and with 3 surfaces (surfaces S1, S2, and S3, dashdotted line) together with the respective ray bundle focus and the wavefront focus. The black dashed, dashdotted and dotted lines indicate the paraxial focus with a 1 surface cornea model (keratometric with S1, keratometer index n_K_ = 1.332), a 2 surface cornea model (S1 and S3), and a 3 surface cornea model (S1, S2, and S3) respectively. The graph shows that the paraxial corneal especially with the cornea model with 1 surface (keratometric power), but also with the cornea models with 2 or 3 surfaces is systematically higher (lower focal distances) compared to the corneal power derived from raytracing.

**Figure 5:**
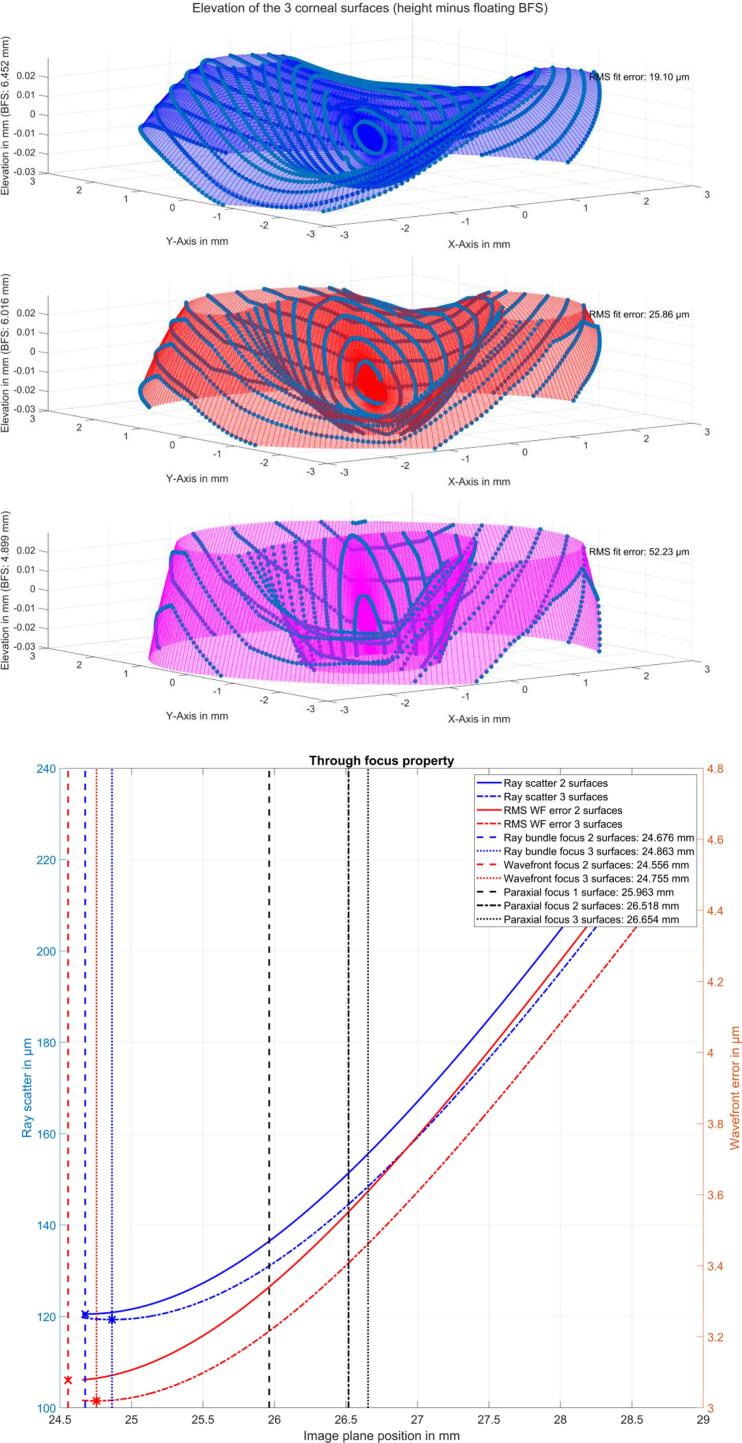
Clinical case C with keratoconus. Upper three plots: Surface elevation as the height difference between the measurement data (filled dots) and the best fit sphere BFS is plotted together with the Gaussian Process (GP) surface representation used for raytracing (semitransparent surface) for the corneal epithelium (surface S1, upper plot), corneal stroma (surface S2, middle plot), and corneal endothelium (surface S3, lower plot). The root mean squared fit error of the GP surface representation is shown in the legend of each plot. Please note that the plots are clipped in the vertical axis to maintain the same axis range as in Figure 3, Figure 4, Figure 6 for better comparability. Lower graph: Through focus performance (centered to the paraxial focus of keratometry with a range of ±2 mm) of the root mean squared ray bundle scatter (blue lines, scale on the left) and the root mean squared wavefront error (red lines, scale on the right) is shown for the corneal model with 2 surfaces (surface S1 and S3, solid line) and with 3 surfaces (surfaces S1, S2, and S3, dashdotted line) together with the respective ray bundle focus and the wavefront focus. The black dashed, dashdotted and dotted lines indicate the paraxial focus with a 1 surface cornea model (keratometric with S1, keratometer index n_K_ = 1.332), a 2 surface cornea model (S1 and S3), and a 3 surface cornea model (S1, S2, and S3) respectively. The graph shows that the paraxial corneal especially with the cornea models with 2 or 3 surfaces, but also with the cornea model with 1 surface (keratometric power) is systematically lower (larger focal distances) compared to the corneal power derived from raytracing.

**Figure 6:**
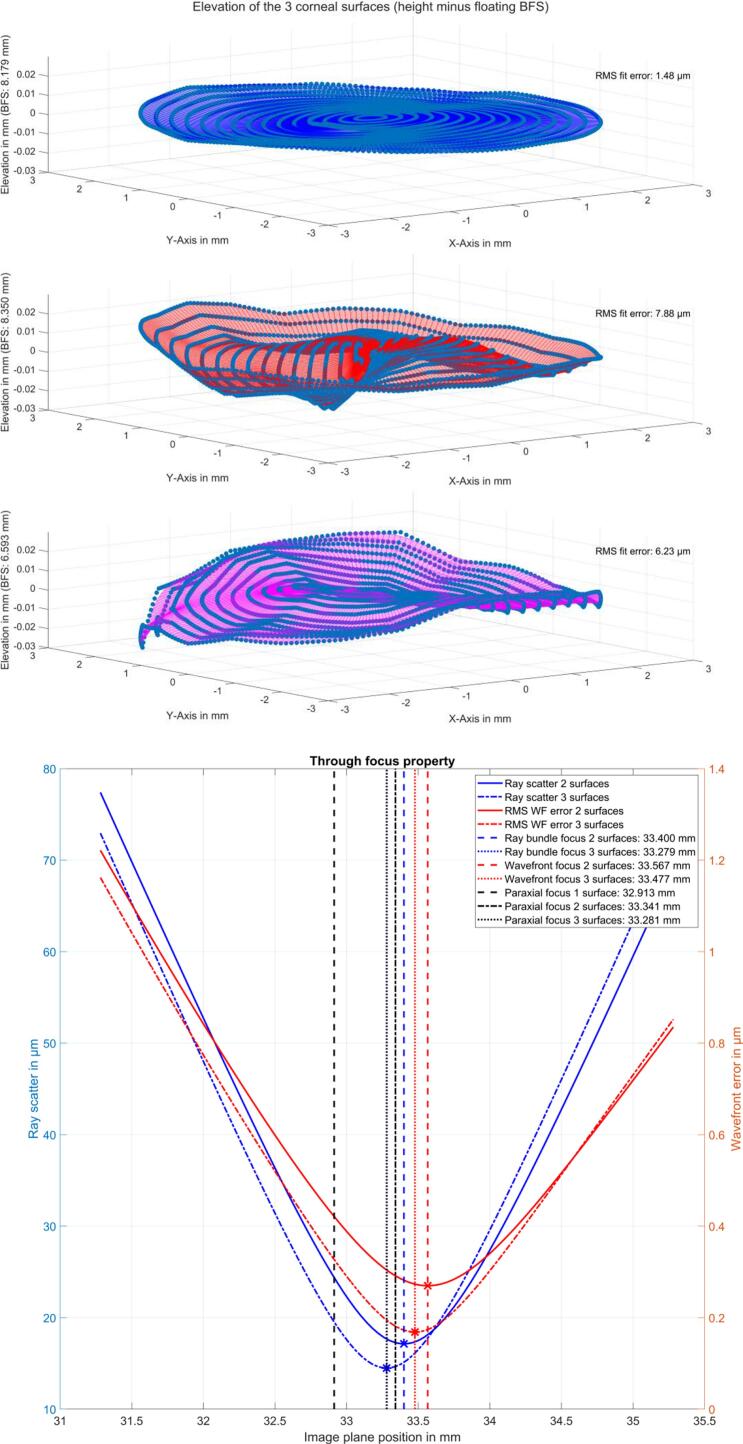
Clinical case D with a situation after laser ablation of the surface with photorefractive keratectomy (PRK) and subsequent Salzmann nodule development. Upper three plots: Surface elevation as the height difference between the measurement data (filled dots) and the best fit sphere BFS is plotted together with the Gaussian Process (GP) surface representation used for raytracing (semitransparent surface) for the corneal epithelium (surface S1, upper plot), corneal stroma (surface S2, middle plot), and corneal endothelium (surface S3, lower plot). The root mean squared fit error of the GP surface representation is shown in the legend of each plot. It is obvious from the graph that especially the stromal surface shows some irregularities. Lower graph: Through focus performance (centered to the paraxial focus of keratometry with a range of ±2 mm) of the root mean squared ray bundle scatter (blue lines, scale on the left) and the root mean squared wavefront error (red lines, scale on the right) is shown for the corneal model with 2 surfaces (surface S1 and S3, solid line) and with 3 surfaces (surfaces S1, S2, and S3, dashdotted line) together with the respective ray bundle focus and the wavefront focus. The black dashed, dashdotted and dotted lines indicate the paraxial focus with a 1 surface cornea model (keratometric with S1, keratometer index n_K_ = 1.332), a 2 surface cornea model (S1 and S3), and a 3 surface cornea model (S1, S2, and S3) respectively. The graph shows that the focus distance from paraxial calculations with the 2 and 3 surface model of the cornea and the focal distance derived with raytraing are close together, whereas the paraxial focal distance of the 1 surface cornea model is smaller, indicating an overestimation of keratometric power.

Figure 3, Figure 4, Figure 5, Figure 6 present results for each of the clinical cases A) to D). In each figure, the upper three plots are surface representations of the corneal epithelium (front surface, upper plot), the corneal stroma (middle plot) and the corneal endothelium (back surface, lower plot) calculated as the elevation with respect to a best fit sphere (BFS radius specified in the Y-label of each plot).

Fig. 3 presents results for clinical case A with a history of hyperopic laser refractive surgery (LASIK). The plots show that the front surface is well represented by a spherical surface, whereas the stromal surface and the corneal back surface show some variation in the azimuthal direction indicating an amount of astigmatism. The smoothed surface representations generated by the GP Predictor show a very small fit error of 2.0 µm for the corneal front surface and larger but still relatively small fit errors for the stromal surface (5.8 µm) and the corneal back surface (7.1 µm). The final plot in Fig. 3 displays the through focus performance for the root mean squared ray scatter and the root mean squared wavefront error. This graph shows that the bundle focus for the cornea models with 2 and 3 surfaces (FRB2 and FRB3), the wavefront focus for the cornea models with 2 and 3 surfaces (FWF2 and FWF3), and the paraxial focus derived from the 1 surface model (FK) and the 2 and 3 surface thick lens cornea models (F2 and F3) all are very close together with a slight underestimation of corneal power with the paraxial setup. The corresponding numerical results are listed in Table 1. Supplementary Fig. A shows the corresponding ray scatter images in the ray bundle focus for the cornea models with 2 (focus at FRB2, left image) and 3 surfaces (focus at FRB3, right image).

**Table 1:** Specifications of the 4 schematic model eyes under test: R_a_ / R_p_ refer to the central corneal front and back surface curvatures, Q_a_ / Q_p_ to the corneal front and back surface asphericities, d_C_ to the central corneal thickness, n_C_ and n_A_ to the refractive indices of the cornea and aqueous humour respectively. The axial position of the aperture stop is provided as the distance from the corneal back surface apex. The Liou-Brennan model eye considers a decentred aperture stop and a tilted incident beam; all other model eyes are strictly centred and coaxial.

**Refractive surfaces, focal distances and corneal power**			**Case A**	**Case B**	**Case C**	**Case D**
Surface characterization with floating BFS and Gaussian Process (GP) prediction models	Epithelium (S1)	Apex X/Y/Z in mm	0.007 0.003 0.001	−0.001 0.033 −0.002	0.046 0.239 0.003	0.020 −0.006 −0.001
		Radius of BFS R1 in mm	7.315	9.331	6.452	8.179
		GP Fit error in µm	2.003	3.486	19.104	1.476
	Stroma (S2)	Apex X/Y/Z in mm	0.012 0.016 0.050	−0.006 0.033 0.070	0.050 0.235 0.045	0.025 −0.006 0.070
		Radius of BFS R2 in mm	7.006	9.441	6.016	8.350
		GP Fit error in µm	5.806	4.214	25.857	7.876
	Endothelium (S3)	Apex X/Y/Z in mm	0.005 0.012 0.538	0.067 0.034 0.474	−0.004 0.195 0.465	0.082 −0.099 0.486
		Radius of BFS R3 in mm	6.179	6.473	4.899	6.593
		GP Fit error in µm	7.100	9.271	52.235	6.226
Paraxial raytracing with 1, 2, and 3 corneal surfaces	Keratometry (S1)	Focal distance FK in mm	29.435	37.547	25.963	32.913
		Corneal power PK in D	45.388	35.582	51.458	40.592
	2 surfaces (S1, S3)	Focal distance F2 in mm	45.147	38.991	26.518	33.341
		Corneal power P2 in D	29.592	34.265	50.381	40.071
	3 surfaces (S1, S2, S3)	Focal distance F3 in mm	45.013	38.945	26.654	33.281
		Corneal power P3	29.680	34.304	50.123	40.143
Raytracing setup with 2 corneal surfaces (S1 and S3)	RMS Ray bundle focus	Focal distance FRB2 in mm	29.137	39.825	24.676	33.401
		RMS ray scatter in µm	18.289	24.725	120.525	17.213
		RMS wavefront error in µm	0.439	0.375	3.976	0.283
	RMS wavefront focus	Focal distance FWF2 in mm	29.106	40.015	24.556	33570
		RMS ray scatter in µm	18.336	25.345	120.671	18.234
		RMS wavefront error in µm	0.439	0.370	3.077	0.271
Raytracing setup with 3 corneal surfaces (S1, S2, S3)	RMS ray bundle focus	Focal distance FRB2 in mm	29.253	39.895	24.863	33.281
		RMS ray scatter in µm	17.528	27.428	119.305	14.562
		RMS wavefront error in µm	0.412	0.387	3.021	0.196
	RMS wavefront focus	Focal distance FWF2 in mm	29.224	40.126	24.755	33.480
		RMS ray scatter in µm	17.567	27.053	119.425	16.217
		RMS wavefront error in µm	0.412	0.386	3.019	0.169

Fig. 4 presents results for case B with a history of myopic laser refractive surgery (LASIK). The plots show that the front surface and the stromal surface are well represented by spherical surfaces, whereas the corneal back surface shows some variation in azimuthal direction indicating a degree of astigmatism. The smoothed surface representations generated by the GP Predictor show small fit errors for all 3 surfaces (front surface: 3.5 µm, stroma: 4.2 µm, back surface: 9.3 µm). The final plot in Fig. 4 displays the through focus performance for the root mean squared ray scatter and the root mean squared wavefront error. This graph shows that the bundle focus for the cornea models with 2 and 3 surfaces (FRB2 and FRB3) and the wavefront focus for the cornea models with 2 and 3 surfaces (FWF2 and FWF3) are close together, but systematically more distant from the corneal apex than the paraxial focus derived from the thick cornea models with 2 or 3 surfaces (F2 and F3) and especially more than the paraxial focus derived from the 1 surface cornea (FK), indicating a systematic overestimation of paraxial (especially keratometric) power. The corresponding numerical results are listed in Table 1. Supplementary Fig. B shows the corresponding ray scatter images in the ray bundle focus for the cornea models with 2 (focus at FRB2, left image) and 3 surfaces (focus at FRB3, right image).

Fig. 5 presents results for case C with a clinical situation of keratoconus. The plots show that all 3 surfaces show a large deviation from the respective floating best fit sphere BFS. Even though the smoothed surface representations generated by the GP Predictor follow the measured datapoints quite well, it is clear from the 3 plots that the Gaussian Process surface representation could not completely follow the noisy measurement data and this is reflected in the relatively large fit errors (front surface: 19.1 µm, stroma: 25.9 µm, back surface: 52.2 µm). The final plot in Fig. 5 displays the through focus performance for the root mean squared ray scatter and the root mean squared wavefront error. This graph shows that the bundle focus for the cornea models with 2 and 3 surfaces (FRB2 and FRB3) and the wavefront focus for the cornea models with 2 and 3 surfaces (FWF2 and FWF3) are close together, but systematically closer to the corneal apex than the paraxial focus derived from the 1 surface cornea (FK) and especially closer than the paraxial focus derived from the thick cornea models with 2 or 3 surfaces (F2 and F3). The corresponding numerical results are listed in Table 1. Supplementary Fig. C shows the corresponding ray scatter images in the ray bundle focus for the cornea models with 2 (focus at FRB2, left image) and 3 surfaces (focus at FRB3, right image).

Fig. 6 presents results for case D with a clinical situation after laser ablation of the surface with photorefractive keratectomy (PRK) and subsequent Salzmann nodule development. The plots show that the corneal front surface (upper plot) is well represented by the floating best fit sphere, whereas the corneal back surface (lower plot) and especially the corneal stroma (middle plot) show some systematically irregular height variation with respect to their best fit spheres. The smoothed surface representations generated by the GP Predictor show a very good performance for the corneal front surface with a root mean squared fit error of 1.5 µm, and larger but still relatively low fit errors for the stromal surface (7.8 µm) and the corneal back surface (6.2 µm). The final plot in Fig. 6 displays the through focus performance for the root mean squared ray scatter and the root mean squared wavefront error. This graph shows that the bundle focus for the cornea models with 2 and 3 surfaces (FRB2 and FRB3) and the wavefront focus for the cornea models with 2 and 3 surfaces (FWF2 and FWF3) as well as the paraxial focus derived from the thick cornea model with 2 or 3 surfaces (F2 and F3) are close together, but slightly more distant from the corneal apex than the paraxial focus derived from the 1 surface cornea (FK). The corresponding numerical results are listed in Table 1. Supplementary Fig. D shows the corresponding ray scatter images in the ray bundle focus for the cornea models with 2 (focus at FRB2, left image) and 3 surfaces (focus at FRB3, right image).

## Discussion

Recent years have seen much discussion about the refractive effect of the epithelium and its consequences e.g. for lens power calculations prior to cataract surgery [1], [2], [5]. Especially following (hyperopic) corneal refractive surgery procedures such as PRK or LASIK, there can be a ‘memory effect’ of the cornea causing some regression of the refractive change after surgery by partially filling in the cornea in the ablation area with epithelium. This study compares raytracing results from two corneal models (the classical model with a single layer and 2 refracting surfaces, and a two-layer model with epithelium and stroma and 3 refracting surfaces) against the corresponding paraxial calculations.

For the study we used tomographic height data for the corneal epithelium, stroma, and endothelium from the MS-39 device and performed a simple spherical fit with a floating sphere and also generated an individual surface representation using a Gaussian Process Predictor surface fit with adaptive kernels derived from a region of interest of 6 mm in diameter. To avoid extrapolation of this GP Predictor, the region of interest should always be at least the size of the bundle of rays passing through the cornea [10]. Assuming a magnification of the real pupil of 15% due to corneal refraction, we should use a surface representation of at least 1.15 times the aperture stop used for raytracing (in our study 1.15·3.5 mm = 4.03 mm).

Two different focus metrics were assessed: the bundle focus with the smallest root mean squared ray scatter, and the wavefront focus with the lowest root mean squared wavefront error. These were evaluated at the paraxial foci calculated using both a simplified single surface corneal model (floating best fit sphere for the corneal front surface S1, comparable to keratometry) and a thick lens model of the cornea with 2 or 3 surfaces (floating best fit spheres for S1 and S3 or S1, S2 and S3) to obtain some insight on the quality of the paraxial simplification.

For the 2 clinical cases after refractive laser surgery (cases A and B) and for the case following laser smoothing (case D) the epithelium seems very smooth and is mostly represented by a sphere, whereas at the corneal back surface some astigmatism (cases A and B) or some irregularity (case D) remains. Comparing the stromal surface and the epithelial surface we learn, especially with case A, but also with case B, that some astigmatism of the stroma could be masked by the epithelium. Case D shows that e.g. after surgical removal of Salzmann nodules the stromal surface could be somewhat irregular, but the epithelium masks most of the stromal irregularities and improves the focal image. However, case C shows that in situations such as in keratoconus there could be tremendous surface irregularities and local variations in surface height. Since the tomographic measurement of the underlaying refractive surfaces (stroma and endothelium) is always affected by the refraction of the corneal front surface (epithelium) there might be some inaccuracy of the tomographic measurement itself [10], [11], [12]. However, the GP Predictor performs surprisingly well for all 3 surfaces, as confirmed in Fig. 5 – although the fit error is systematically larger in case C than in the other clinical cases, the individual surface representation follows the irregular measurement data points quite accurately, whereas a spherical surface model (the floating best fit sphere) seems to be insufficient.

In addition our results show – with restrictions to our 4 clinical cases – that there is no systematic and clinically relevant impact of the epithelium on corneal refraction derived from raytracing. After hyperopic LASIK the foci of raytracing with either 2 or 3 surfaces match the respective foci of the paraxial calculations with 1, 2 or 3 refractive surfaces quite well. In contrast, after myopic LASIK the raytracing foci are quite consistent but located far away from the paraxial focus especially with a simple 1 surface model and even with a 2 or 3 surface model, indicating an underestimation of corneal power when using paraxial calculations. Furthermore, even in clinical example C (keratoconus), the ray bundle focus and the wavefront focus derived with raytracing based on a 2 or 3 surface cornea model match surprisingly well, whereas the paraxial foci derived from a floating best fit sphere systematically overestimate the focal distance and underestimate the corneal power.

There is currently no consensus in the literature about which focus metric to optimise. Typical metrics include the root mean squared scatter of the rays in the image space, the overall diameter of the rays intersecting a surface orthogonally to the chief ray, or the root mean squared wavefront error derived from the optical pathlength differences of all rays from the source to the image considered at the aperture plane. The focus metric directly affects the position of the best focus as shown in the through focus performance curves (lower graphs of Figure 3, Figure 4, Figure 5, Figure 6).

However, our study shows some limitations: firstly, the refractive index of the epithelium and maybe also the stroma is not fully representative [1], [4], [5] and should be verified by new experiments. Secondly, to avoid complexity, we used a fixed pupil size of 3.5 mm located 3.66 mm behind the corneal apex, and other pupil size, location, and geometry may produce differing results. When considering large pupils we have to adapt the zone for the surface fit to avoid extrapolation. Thirdly, we used a collimated entrance beam corresponding to a light source at infinity and aligned to the instrument axis of the tomographer. The results could differ to a certain extent for objects or point light sources at finite distances with a divergent entrance beam at the cornea [8]. Fourthly, after evaluating several closed representations for refractive surfaces, we selected a Gaussian Process Predictor model for the refractive surfaces of the cornea as trade-off between individuality and smoothness. Fifthly, there are no literature data about noise or inaccuracies in tomographic measurements [23] of the corneal epithelium. And finally, this raytracing strategy should be tested with a large dataset of tomographic examinations to verify the general applicability.

**In conclusion**, in this paper we investigated refraction of the cornea based on raytracing using tomographic measurement data of the corneal epithelium, stroma, and endothelium. A customized representation of the 3 refracting surfaces was developed based on Gaussian Process representations which appear to be a good trade-off between global and local surface representations, providing a good balance between the smooth surface required for raytracing and the retention of genuine surface detail. The raytracing results with the classical 2 surface model (epithelium and endothelium) and the 3 surface model (epithelium, stroma, and endothelium) were evaluated and compared to the results of a paraxial calculation based on simplified spherical surface models of the corneal epithelium (equivalent to keratometry), and thick lens representations of the cornea with 2 or 3 spherical surfaces. On the basis of our 4 clinical examples we feel that the corneal stroma has a minor impact on the refractive power and imaging property of the cornea, but in special clinical situations raytracing might yield different results than paraxial calculations.

## CRediT authorship contribution statement

**Achim Langenbucher:** Writing – original draft, Validation, Project administration, Methodology, Investigation, Formal analysis, Conceptualization. **Nóra Szentmáry:** Validation, Supervision, Investigation. **Alan Cayless:** Writing – original draft, Project administration, Methodology, Formal analysis. **Peter Hoffmann:** Supervision, Project administration, Methodology, Formal analysis. **Jascha Wendelstein:** Writing – original draft, Validation, Project administration, Methodology, Formal analysis, Data curation.

## Declaration of competing interest

The authors declare the following financial interests/personal relationships which may be considered as potential competing interests: The author is an Editorial Board Member/Editor-in-Chief/Associate Editor/Guest Editor for this journal and was not involved in the editorial review or the decision to publish this article

AL: Speakers fees from Hoya Surgical and Johnson & Johnson unrelated to the presented material; NS & AC: None; PH: Speakers fees from Heidelberg Engineering, Hoya Surgical and Johnson & Johnson unrelated to the presented material; JW: speakers fees from Hoya Surgical, Johnson & Johnson and Carl-Zeiss-Meditec unrelated to the presented material.
