# Supplementary material for: Impact of the corneal epithelium on the corneal power using 3D raytracing with OCT data
Source: Z Med Phys. 2025 Feb 11;36(2):157–68. doi: 10.1016/j.zemedi.2025.01.002 (PMC13316403; doi:10.1016/j.zemedi.2025.01.002)
Supplement: Supplementary Data 1 [file mmc1.docx]

**Supplementary Figure**


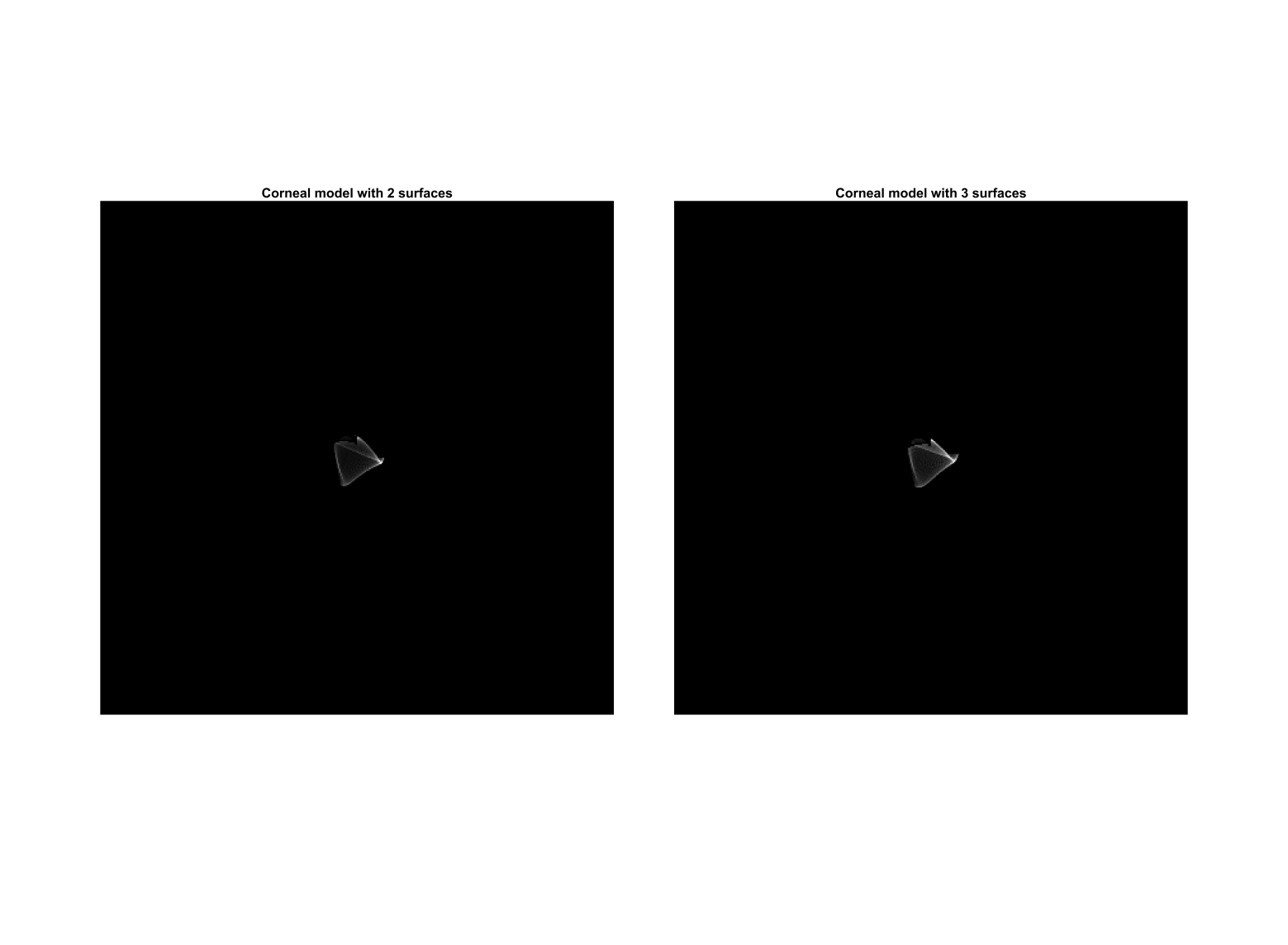
Subfigure A


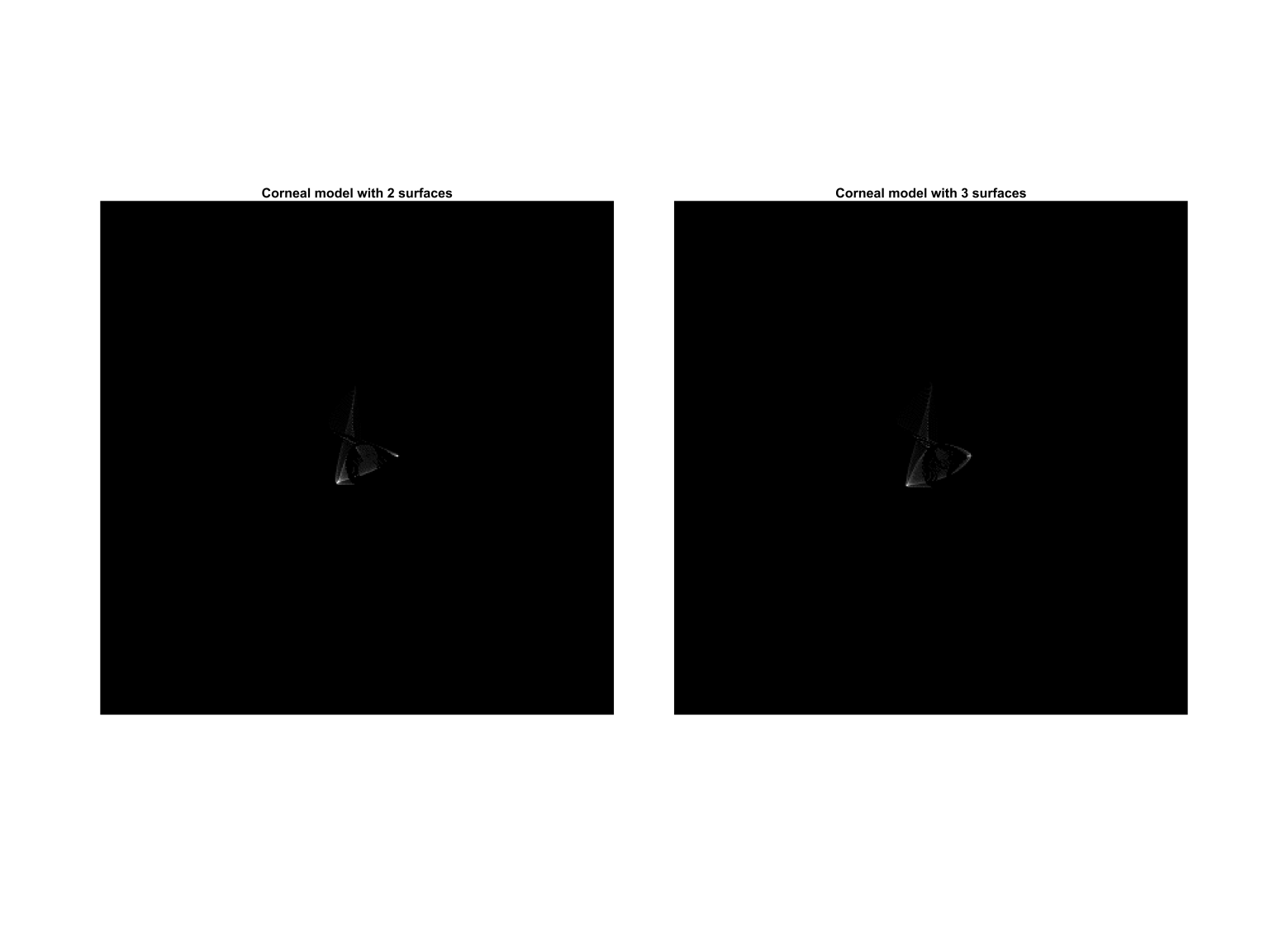
Subfigure B


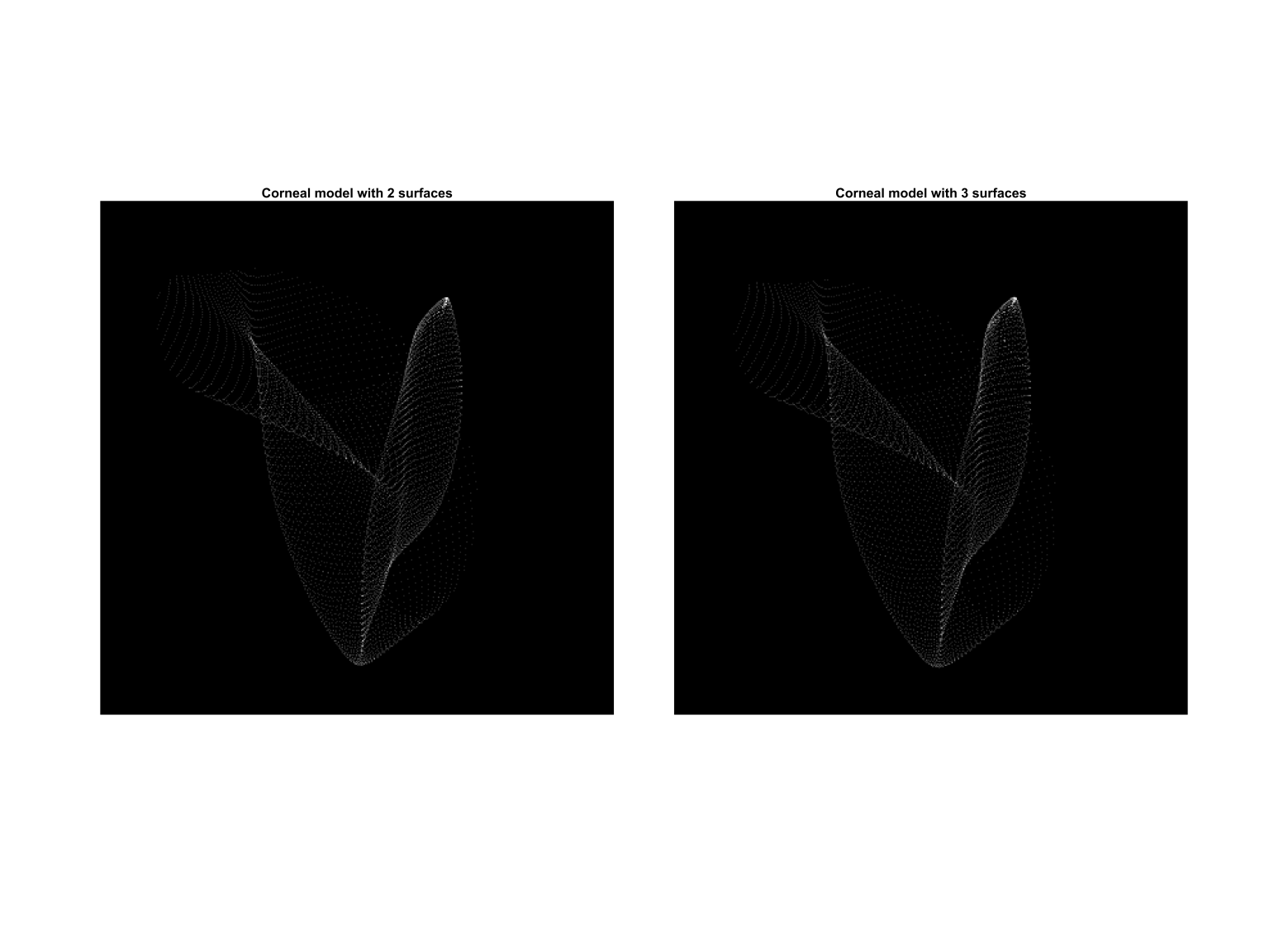
Subfigure C


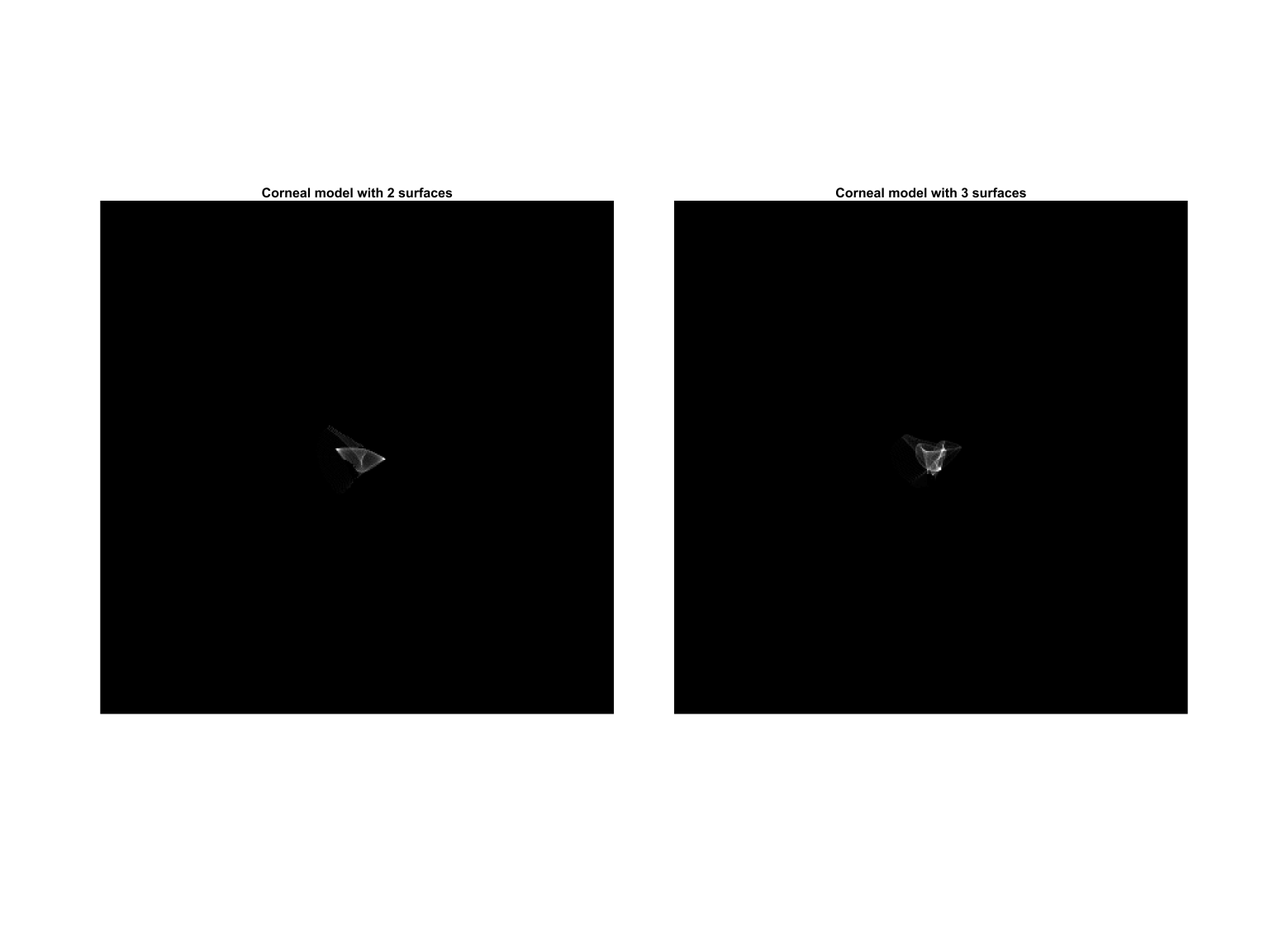
Subfigure D

**Supplementary Figure**: Ray scatter images at the bundle focus plane (focus at FRB2) derived from raytracing through the cornea with 2 surfaces (corneal front surface S1 and corneal back surface S3, left images), and the corresponding rayscatter images at the bundle focus plane (focus at FRB3) from the cornea considered with 3 surfaces (corneal front surface S1 corneal stroma S2, and corneal back surface S3, right images). Subfigures refer to: situation after hyperopic refractive laser surgery (case A, subfigure A), situation after myopic refractive laser surgery (case B, subfigure B), situation with keratoconus (case C, subfigure C), and situation after laser ablation of the surface with photorefractive keratectomy and subsequent Salzmann nodule development (case D, subfigure D). The image size refers to a dimension of 400x400 µm at focal plane.
